# Alterations in cardiac function correlate with a disruption in fatty acid metabolism in a mouse model of SMA

**DOI:** 10.1093/hmg/ddaf006

**Published:** 2025-01-15

**Authors:** Nithya N Nair, Rachel A Kline, Imogen Boyd, Meenakshi Anikumar, Adrian Thomson, Douglas J Lamont, Gillian A Gray, Thomas M Wishart, Lyndsay M Murray

**Affiliations:** Centre for Discovery Brain Sciences, Hugh Robson Building, George Square, University of Edinburgh, Edinburgh EH8 9XD, United Kingdom; Euan McDonald Centre for Motor Neuron Disease Research, Hugh Robson Building, George Square, University of Edinburgh, Edinburgh EH8 9XD, United Kingdom; Euan McDonald Centre for Motor Neuron Disease Research, Hugh Robson Building, George Square, University of Edinburgh, Edinburgh EH8 9XD, United Kingdom; The Roslin Institute, Royal (Dick) School of Veterinary Studies, The University of Edinburgh, Easter Bush Campus, Midlothian EH25 9RG, United Kingdom; Centre for Discovery Brain Sciences, Hugh Robson Building, George Square, University of Edinburgh, Edinburgh EH8 9XD, United Kingdom; Centre for Discovery Brain Sciences, Hugh Robson Building, George Square, University of Edinburgh, Edinburgh EH8 9XD, United Kingdom; Centre for Cardiovascular Science, The Queen's Medical Research Institute, 47 Little France Crescent, The University of Edinburgh, Edinburgh EH16 4TJ, United Kingdom; FingerPrints Proteomics Facility, School of Life Sciences, Dow Street, University of Dundee, Dundee DD1 5EH, United Kingdom; Centre for Cardiovascular Science, The Queen's Medical Research Institute, 47 Little France Crescent, The University of Edinburgh, Edinburgh EH16 4TJ, United Kingdom; Euan McDonald Centre for Motor Neuron Disease Research, Hugh Robson Building, George Square, University of Edinburgh, Edinburgh EH8 9XD, United Kingdom; The Roslin Institute, Royal (Dick) School of Veterinary Studies, The University of Edinburgh, Easter Bush Campus, Midlothian EH25 9RG, United Kingdom; Centre for Systems Health and Integrated Metabolic Research, Clifton Boulevard, Nottingham Trent University, Nottingham NG1 4GG, United Kingdom; Centre for Discovery Brain Sciences, Hugh Robson Building, George Square, University of Edinburgh, Edinburgh EH8 9XD, United Kingdom; Euan McDonald Centre for Motor Neuron Disease Research, Hugh Robson Building, George Square, University of Edinburgh, Edinburgh EH8 9XD, United Kingdom

**Keywords:** heart, spinal muscular atrophy, proteomics, lipid, metabolism

## Abstract

Spinal Muscular Atrophy is an autosomal dominant disease caused by mutations and deletions within the *SMN1* gene, with predominantly childhood onset. Although primarily a motor neuron disease, defects in non-neuronal tissues are described in both patients and mouse models. Here, we have undertaken a detailed study of the heart in the *Smn^2B/−^* mouse models of SMA, and reveal a thinning of the ventriclar walls as previously described in more severe mouse models of SMA. However most structural changes are resolved by accounting for the smaller body size of the SMA mouse, as was also confirmed in the SMN∆7 model. Echocardiography revealed increased systolic function, which was particularly pronounced in subsets of mice and an increase in global longitudinal strain, collectively indicative of increased cardiac stress in the *Smn^2B/−^* mouse model. We have used TMT proteomics to perform a longitudinal study of the proteome of the hearts of *Smn^2B/−^* mice and reveal a progressive dysregulation of LXR/RXR signalling which is a regulator of lipid metabolism. We further show consistent perturbations in lipid metabolism in the *Smn^2B/−^*, *Smn−/−;SMN2;SmnΔ7*and *Smn^Δ7/Δ7^;SMN2* mouse models of SMA on the day of birth. This work indicates that although structural changes in the heart can be overstated by failing to account for body size, there are functional defects which could predispose the heart to subsequent failure. We identify a common molecular signature across mouse models pointing to a dysregulation in lipid metabolism, and suggest that manipulation of LXR/RXR signalling offers an opportunity to impact upon these pathways.

## Graphical Abstract

**Graphical Abstract:**
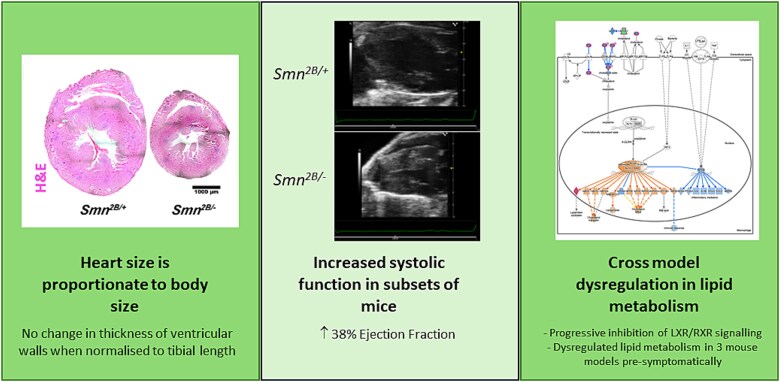

## Introduction

Spinal Muscular Atrophy (SMA) is caused by mutations and deletions within the survival motor neuron 1 *(SMN1)* gene causing a motor neuron disease with childhood onset. SMA is clinically classified into different types (I-IV) which vary in their severity, with the most important modifier being copy number of the telomeric copy of the gene termed survival motor neuron 2 *(SMN2)*. Three treatments have now been approved for SMA which all aim to increase functional SMN levels [1–4]. Whilst these treatments have transformed the prognosis for patients who are diagnosed with SMA, there is an ongoing need to understand the pathogenesis of SMA and gain a comprehensive understanding of what defects could persist or manifest later, even after treatment with an SMN upregulating therapy [5, 6].

Whilst traditionally considered a motor neuron disease, there is increasing evidence for the involvement of non-neuronal tissues. Indeed, congenital heart defects such as atrioseptal defects, ventriculoseptal defects and hypoplastic heart syndrome have been described in patients with genetically confirmed SMA [7], with a particularly high incidence of congenital heart defects in those with the lowest copy numbers of *SMN2* [8, 9]. However, perturbed left atrium function and bradycardia has also been observed in patients with type II and III SMA [10]. An increase in global longitudinal strain, a measure of cardiac stress, has been reported to be perturbed in children with type II and III SMA [11]. Subsequent work in the Smn−/−;SMN2;SmnΔ7 (jax mouse line005025; herein referred to as SMNΔ7) and Smn^Δ7/Δ7^;SMN2 (Jax mouse line 005058; herein referred to as the Taiwanese) mouse model of SMA has revealed structural defects consistent with patient abnormalities, such as thinning of the interventricular septum and left ventricular wall, and dilation of the ventricles [12–17]. Concurrent findings in mouse models include fibrosis, reduced cell density, decreased microvascular density, decreased proliferation and decreased elastin levels within the myocardium [14, 18–20]. *In vivo* imaging has also revealed functional changes such as decreased heart rate, ejection fraction, stroke volume and cardiac output [12, 13, 17]. It is notable that many defects were progressive over a similar time-course as the motor neuron pathology, and were not present embryonically or at birth [14, 19, 21]. Indeed, disrupted sympathetic innervation to the heart has been proposed as a possible mechanism [13]. However, primary cultures of cardiomyocytes from an SMA mouse model and iPSC derived cardiomyocytes display altered contractile dynamics and perturbed calcium handling which was corrected by the addition of Smn [20, 22]. This is indicative of a requirement for Smn in the heart specifically. While administration of Smn upregulating compounds in mouse models appears to correct some anomalies, reductions in functional parameters such as stroke volume, cardiac output, left ventricular mass, heart rate and ejection fraction persisted even, after early treatment [12, 17]. This presents the possibility that heart defects will remain in SMA patients following treatment, and underlines the importance of a comprehensive understanding of the underlying basis of heart pathology in SMA.

From the above, it is evident that heart defects are present in at least a subset of patients with SMA. There are also consistent heart defects in severe mouse models of SMA, however it is currently unclear how representative these are of the defects in patients. Although there is a clear correlation between the progression of heart defects and motor neuron pathology in mouse models, it is unclear if and how they are related. An additional important consideration is that mouse models of SMA show a progressive and significant decrease in relative body size compared to healthy control littermates. This is likely to impact upon the physical size of the heart and morphological measurement taken from the heart, and will also have an impact upon function, with a reduction in blood volume. Whilst there are instances where metrics such as heart weight have been normalised to surrogates for body size (body weight/tibial length/femoral length; [13–15, 19, 20]), the majority of other structural and functional measures rarely account for the body size. The extent to which reduced body size impacts upon data describing heart defects in SMA mouse models is therefore unclear.

The majority of work looking at heart defects in mouse models of SMA has been performed on relatively severe mouse models, with a life expectancy of 10–12 days and often displaying motor neuron pathology by P2. The *Smn^2B/−^* mouse model is also a severe mouse model of SMA; however, as it is less severe than the SMN∆7 mouse model or the Taiwanese mouse model, it offers a more protracted disease time course with a clear pre-symptomatic window until P10, followed by a clear degenerative phenotype [23, 24]. This model therefore offers the opportunity to gain insight into the initiating mechanisms underlying pathogenesis in SMA and offers a protracted period to analyse the progression of pathology.

Here, we have undertaken a detailed structural, functional and molecular study of the hearts in *Smn^2B/−^* mice. At disease end-stage, we describe similar structural changes within the heart as has been previously reported, but reveal that the majority of structural anomalies are resolved by accounting for the decrease in body size. *In vivo* imaging revealed an increase in ejection fraction, which was particularly pronounced in two out of three cohorts of mice. There was also an increase in global longitudinal strain consistent with underlying cardiac stress in all cohorts of mice. To gain insight into the mechanisms underlying this cardiac stress, we performed proteomic analysis which revealed a large number of proteins and pathways which are progressively altered between P0 and P18. Specifically, the highest z scores and lowest P values were associated with LXR/RXR signalling, a pathway which regulates lipid metabolism. We further performed comparative proteomic analysis across 3 mouse models of SMA and reveal a consistently predicted dysregulation of lipid metabolism at P0. Collectively, this work gives insight into the mechanisms which may underlie cardiac stress in SMA and putative pathways which could be therapeutically targeted.

## Materials and methods

### Mouse maintenance

*Smn^2B/2B^* mice [22; C57Bl/6J congenic background] were interbred with *Smn^+/−^* (Jackson Laboratories strain formerly 010921, now 006214, C57Bl/6 congenic background) to obtain *Smn^2B/+^* control mice and *Smn^2B/−^* experimental mice. *Smn^+/^;SMN2^+/+^;SMNΔ7^+/+^* (Jackson strain 005025, FVB congenic background) mice were interbred to produce *Smn^−/−^;SMN2^+/+^;SMNΔ7^+/+^* (aka *SMNΔ7*) mice. The FVB.Cg-*Smn1^tm1Hung^*Tg(SMN2)2Hung/J mice (Jackson Laboratories strain 005058, FVB background) also known as the *Smn*^−/−^;*SMN2^tg/tg^* mice were crossbred with the heterozygous *Smn^+/−^* mice to generate the *Smn*^−/−^;*SMN2^tg/+^* (aka Taiwanese) mice and the control *Smn*^+/−^;*SMN2^tg/+^* mice (access to these mice were provided by generous agreement with Prof Thomas Gillingwater). All mice were maintained on a 12 h light/dark cycle under pathogen-free conditions within animal facilities of the University of Edinburgh. All procedures were performed in accordance with the UK Home Office. Mice were genotyped using standard PCR protocols. Mice will killed by overdose of anaesthetic and death was confirm by exsanguination via the carotid artery or cervical dislocation.

### Histological and Immunofluorescent staining

Beating hearts were dissected and placed in 30 mM KCl solution for 15 min, for the hearts to be arrested at diastole. Hearts were fixed in 4% paraformaldehyde overnight at 4°C before embedding in paraffin wax and subsequent sectioning. A hind limb was also removed and fixed and a vernier calliper was used to measure tibial length. Serial sections (8 microns) were collected, dewaxed and stained using haematoxylin and eosin using standard protocols.

For Picosirius Red staining, slides were stained in Weigert’s picrosirius red solution (1% Sirius red F3B a saturated 1.3% aqueous picric acid solution) for 60 min in the dark. Slides were dehydrated in ascending alcohol series, cleared twice in xylene and mounted in xylene.

For Oil-Red-O staining, following fixation, hearts were embedding in Optimum Cutting Temperature compound (OCT) and sectioned using a Leica cryostat at 10 μm. The slides were submerged in 60% isopropanol solution then immersed in freshly prepared Oil Red O working solution for 15–20 min. Sections were mounted using an aqueous mounting medium and imaged within 48 h.

For immunofluorescent staining, heat-induced antigen retrieval (HIAR) was performed in 0.01 M sodium citrate solution for IB4 staining. Slides were blocked in 10% goat serum (for IB4) or 1% TritonX100 4% BSA (for Ki67) in PBS for 60 min before addition of primary antibody (biotinylated isolectin B4, I21414, Life Technologies, 1:100; Rabbit anti-Ki67 antibody, Abcam, Cat no ab15580, 1:200) overnight at 4°C and visualised using secondary antibody (1:100, Streptavidin Alexa-488, S32354, Life Technologies; Donkey anti-rabbit IgG, Invitrogen ThermoFisher scientific, Cat no A-21206, 1:500). Rhodamine-conjugated wheat germ agglutinin was applied where required to label the cell membrane (1:75, RL-1022, Vector Laboratories). Slides were exposed to 4′, 6-diamidino-2-phenylindole (DAPI; Life Technologies; 1:1000) and mounted in MOWIOL mounting media (Sigma).

### Quantification of tissue sections

For structural measurements, The Leica Application Suite X (LASX) with an inverted microscope was used to capture overlapping images of the tissue section under 10× magnification and images were montaged together using Adobe Photoshop (Version 21.1.3, Adobe Creative Cloud). The width of the left ventricular wall and the interventricular septum was obtained by taking the average of three repeated measures in the 160–180^0^ and 340–360^0^ respectively. The area measurements were obtained by drawing around the respective regions of interest. The area without the lumen was calculated by adding both the ventricular lumen areas and then subtracting it from the total area occupied by the tissue section.

The nuclear density counts were obtained by overlaying a central grid layout with each of the squares occupying an area of 10 000 μm^2^ and counting the number of nuclei in each 10 000 μm^2^ square.

For cell proliferation, the total number of Ki67 positive cells in 5 fields of view from 3 sections per heart were quantified and normalised to the corresponding cell density count and averaged.

For cardiomyocyte cross sectional area, heart sections were stained with wheat germ agglutinin to mark membrane; IB4 to mark endothelial cells, and DAPI to label nuclei. The blood vessels were identified as double positive for IB4 and WGA. The sections were imaged using the confocal Nikon A1R microscope. In order to quantify cardiomyocytes in cross section, the cardiomyocytes selected were required to have a roundness score of between 0.7 and 1.0, have a central nucleus and be situated adjacent to a blood vessel indicated by IB4. The cross-sectional area of approximately 70–100 cardiomyocytes was measured by drawing each cardiomyocyte across the five images using ImageJ.

To quantify collagen levels, following Picrosirius Red staining, images were captured using the Nikon Ti microscope and stitched together using the NIS Elements software tool. The ‘colour deconvolution’ tool in ImageJ was used to isolate Picosirius red and the ImageJ ‘Threshold’ function was used to obtain the percentage area of each of these regions. The total percentage area of the tissue was divided by the percentage area of picosirius red positively stained tissue area to provide the percentage area of picrosirius red-positive staining (red) relative to the amount of tissue.

To quantify fat levels, the images of the Oil-Red-O stained sections were captured using the Nikon Ti microscope under consistent light and exposure levels and stitched together using the NIS Elements software tool. The threshold was adjusted to highlight the area of the tissue stained by Oil Red O without saturating the signal. Approximately 3–5 sections were captured per sample and raw integrated density was quantified in ImageJ (RawIntDen). The average RawIntDen was calculated across sections for each animal.

### Echocardiography

General anesthesia was induced using isofluorane (induction 4% in 100% O2, then maintained at 1%–2%) and placed supine on a heated monitoring imaging table. All four paws were taped along with Parker Spectra 360 Electrode Gel (PARKER LABORATORIES, INC.Fairfield, USA) down to table ECG electrodes to monitor ECG, respiration and heart rate (450-550 bpm). Body temperature was measured using a rectal probe and maintained at 37°C ± 0.5°C. Any thoracic region hair was removed using depilation cream (Nair hair removal cream, Church & Dwight) and Parkers Aquasonic 100 ultrasound gel was applied before imaging. Echocardiography measurements were acquired using a VisualSonics Vevo 3100 high frequency ultrasound imaging system (FUJIFILM VisualSonics, Inc. Yonge Street, Toronto, Canada) with a MX550D transducer (centre transmit frequency: 40 MHz). EKV (echo kilohertz visualisation) imaging of the Left ventricle (LV) was acquired in the parasternal long axis view (PSLA) and M mode images in the parasternal short axis LV midpoint of with papillary muscles visible at 2 and 4 o’clock position to enable LV cardiac functional measurements. PLAX views were analysed for the strain measurements with Visualsonics VevoStrain software.

Images were analysed blind to experimental group and analysed using the Visualsonics VevoLab 3.1.1 image analysis software.

### Proteomics

Dissected hearts were placed in an M tube with of NP-40 lysis buffer and protease cocktail inhibitor. Samples were homogenised using a gentleMACS dissociator. The homogenate was centrifuged (20 000 × *g* for 20) for 20 min and the supernanant isolated to produce the protein-rich soluble fraction. The protein concentration for each of the samples was determined by using the micro-BCA kit (23 235, Thermo Scientific) for colourimetric detection. For proteomics, hearts from 3 mice were pooled using 50 μg of protein per sample. Tandem mass tagging liquid chromatography mass spectrometry (TMT-LC–MS) was performed at the FingerPrints Proteomics Facility, University of Dundee, UK, as previously described (Ledahawsky et al 2022 PMID: 35092170; Nelvagal et al 2020 PMID: 32938982). Protein samples were trypsinized and desalted at room temperature. 100 μg of desalted tryptic peptides per sample were dissolved in 100 μl of 100 mM Triethylammonium bicarbonate. The 10 different tandem mass tag (TMT) labels comprising the TMT10plex kit (Thermo Fisher Scientific) were dissolved in 41 μl anhydrous acetonitrile. Each dissolved label was added to a different sample— P0 *Smn^2B/−^* (Tag 126), P10 *Smn^2B/−^* (Tag 127 N), P18 *Smn^2B/−^* (Tag 127C), P0 *Smn^2B/+^* (Tag 129N), P10 *Smn^2B/+^* (Tag 129C), P18 *Smn^2B/+^* (Tag 128N), P0 SMN∆7 (Tag 128C), P0 WT (for SMN∆7) (Tag 130N), P0 Taiwanese (130C), and P0 WT (for Taiwanese) (Tag 131). The sample-label mixture was incubated for 1 h at room temperature. Labelling reaction was stopped by adding 8 μl of 5% hydroxylamine per pooled sample.

As previously described, following labelling with TMT, pooled samples were desalted, and dried in a speed-vac at 30°C, re-dissolved in 200 μl ammonium formate (10 mM, pH 10) and peptides were fractionated using an Ultimate 3000 High Performance Liquid Chromatography column (Thermo-Scientific) containing an XBridge C18 column (XBridge peptide BEH, 130 Å, 3.5 μm, 2.1 × 150 mm) (Waters, Ireland) with an XBridge guard column (XBridge, C18, 3.5 μm, 2.1 × 10 mm) (Waters, Ireland). Buffers A (10 mM ammonium formate in milliQ water) and B (10 mM ammonium formate with 90% acetonitrile) were adjusted to pH 10 with ammonia. Fractions were collected using a WPS-3000FC auto-sampler (Thermo-Scientific) at 1 min intervals. Column and guard column were equilibrated for 20 min at a constant flow rate of 0.2 ml/min. 175 μl per sample was loaded onto the column at a rate of 0.2 ml/min, and the separation gradient was started 1 min after sample was loaded onto the column. Peptides were eluted from the column with a gradient of 2%–5% Buffer B in 6 min, and then from 5% to 60% Buffer B in 50 min. The column was washed for 16 min in Buffer B and re-equilibrated at 2%. The fraction collection started 1 min after injection and stopped after 80 min (total 80 fractions, 200 μl each). The total number of fractions concatenated was set to 15 and the content of the fractions was dried and suspended in 50 μl of 1% formic acid prior to analysis with LC–MS/MS.

LC–MS/MS analysis Liquid chromatography–tandem mass spectrometry was performed by FingerPrints Proteomics Facilities at the University of Dundee. But briefly, analysis of peptide readout was performed on a Q Exactive HF Hybrid Quadrupole-Orbitrap Mass Spectrometer (Thermo Scientific) coupled with a Dionex Ultimate 3000 RS (Thermo Scientific). LC buffers were made up to the following: Buffer A [2% acetonitrile and 0.1% formic acid in Milli-Q water (v/v)] and Buffer B (80% acetonitrile and 0.08% formic acid in Milli-Q water (v/v). Aliquots of 15 μl per sample were loaded at a rate of 5 μl/min onto a trap column (100 μm × 2 cm, PepMap nanoViper C18 column, 5 μm, 100 Å, Thermo Scientific) which was equilibrated with 98% Buffer A. The trap column was washed for 6 min at the same flow rate and then the trap column was switched in-line with a resolving C18 column (Thermo Scientific) (75 μm × 50 cm, PepMap RSLC C18 column, 2 μm, 100 Å). Peptides were eluted from the column at a constant flow rate of 300 nl/min with a linear gradient from 95% Buffer A to 40% Buffer B in 122 min, and then to 98% Buffer B by 132 min. The resolving column was then washed with 95% Buffer B for 15 min and re-equilibrated in 98% Buffer A for 32 min. Q Exactive HF was used in data dependent mode. A scan cycle was comprised of a MS1 scan (m/z range from 335 to 1800, with a maximum ion injection time of 50 ms, a resolution of 120 000 and automatic gain control (AGC) value of 3 × 106) followed by 15 sequential-dependent MS2 scans (with an isolation window set to 0.4 Da, resolution at 60 000, maximum ion injection time at 200 ms and AGC 1 × 105. To ensure mass accuracy, the mass spectrometer was calibrated on the first day that the runs were performed.

Protein identification Raw MS data were searched against mouse (*Mus musculus*) protein sequences from UniProtKB/Swiss-Prot using the MASCOT search engine (Matrix Science, Version 2.2) through Proteome Discoverer software (Version 1.4, Thermo Fisher). Parameters for database search were as follows: MS1 Tolerance: 10 ppm; MS2 Tolerance: 0.06da; fixed modification: Carbamidomethyl (C) Variable Modification: Oxidation (M), Dioxidation (M), Acetyl (N-term), Gln- > pyro-Glu (N-term Q), TMT 10(N-term and K); maximum missed cleavage: 2; and target FDR 0.01. All identifications were quantified as relative ratios of expression of SMA to their respective controls at each time point for each tissue. Relative abundance ratios along with UnitProtKB/Swiss-Prot identifications were exported into Microsoft Excel as a raw data file containing ID, ratio of change in expression at each time point.

### Statistical analysis

All experimental groups consisted of a minimum of three animals. Power analysis was performed using G*Power software (Version 3.1.9.4). All results are presented as mean ± standard deviation (SD). P value was determined by the unpaired two-tailed t test or the Mann–Whitney U test and carried out using GraphPad PRISM (Version 10.0.0), where ns not significant, ^*^P < 0.05, ^*^^*^P < 0.01, ^***^P < 0.001 and ^*^^*^^*^^*^P < 0.0001.

## Results

### Structural changes in the hearts of mouse models of SMA are resolved by accounting for body size

**Figure 1:**
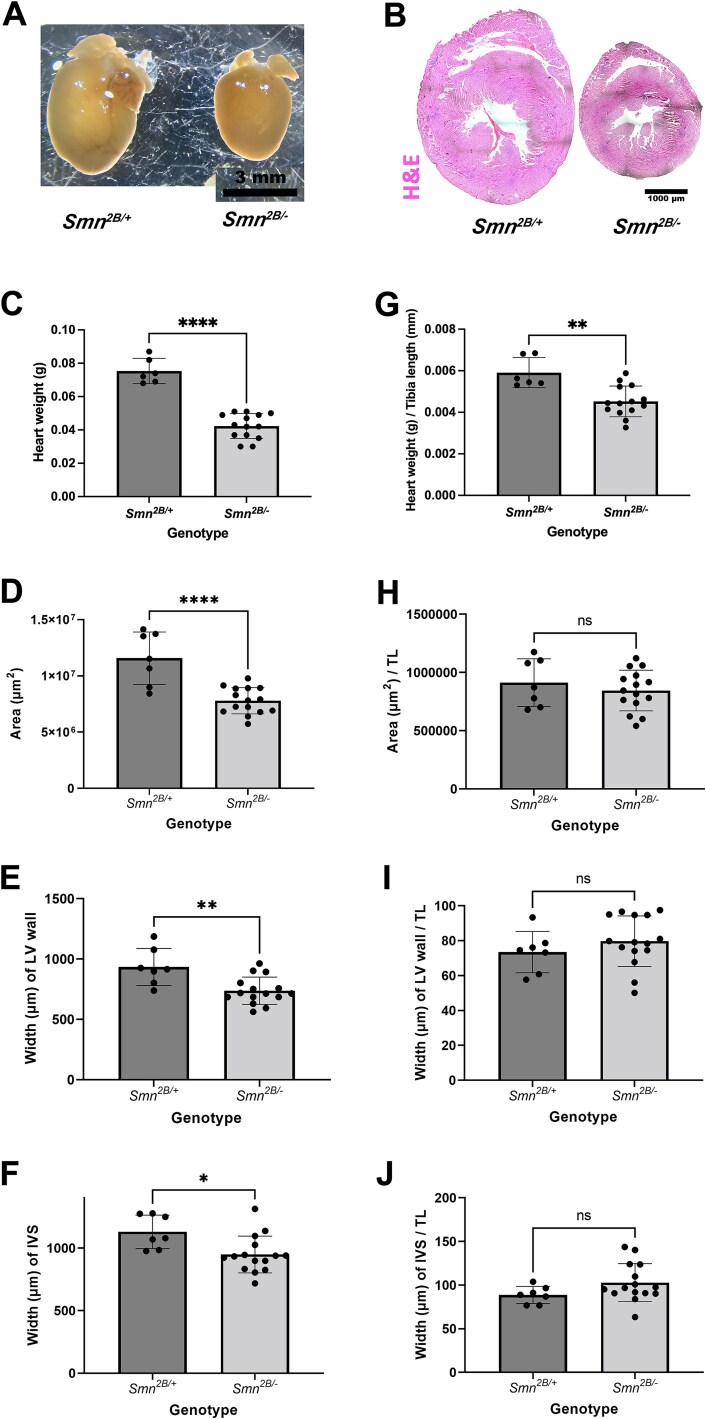
Hearts from P18 *Smn^2B/−^* mice are lighter but otherwise in proportion to body size. (A) Images showing gross morphology of hearts from *Smn^2B/−^* and *Smn^2B/+^* control mice (scale bar = 3 mm). (B) Haematoxylin and eosin stained horizonal sections of hearts at the level of the anterior papillary muscle from P18 *Smn^2B/−^* and *Smn^2B/+^* mice. Scale bar = 1 mm. (C–J) Bar charts showing heart weight (C and G), cross sectional area (D and H), left ventricular (LV) wall width (E and I) and width of the interventricular septum (IVS) (F,J) which are either raw values (C–F) or normalised to tibia length (TL, G–J). ns, not significant; ^*^P < 0.05; ^*^^*^P < 0.01; ^*^^*^^*^^*^P < 0.0001 by the two tailed unpaired T-test. n = 7 hearts for *Smn^2B/+^* and n = 15 hearts for *Smn^2B/−^*).

Previous work has described structural changes in the hearts of mouse models of SMA, including a decrease in heart volume and a thinning of ventricular walls. In order to determine whether these defects were present in the hearts of *Smn^2B/−^* mice, we compared heart weight, size and ventricular wall thickness at P18 (disease end-stage) to healthy control littermates (*Smn^2B/+^*). Hearts from *Smn^2B/−^* mice appeared smaller at a gross level and there was a significant decrease in weight and cross-sectional area (Fig. 1A–D). There was also a significant reduction in the width of the left ventricular wall and interventricular septum, supporting previous findings in other models (Fig. 1E and F) [12–17]. However, since the overall size of the heart is smaller, it is possible that the decreases observed are consistent with body size. To account for this, all measurements were normalised to the tibia length, which is a proxy for body size. Tibia length was chosen over body weight due the possible contributions of muscle atrophy to confound body weight measurements. As expected, tibia length was significantly smaller in *Smn^2B/−^* mice compared to *Smn^2B/+^* mice (Mean ± SEM: 9.39 ± 1.32 vs 12.73 ± 0.22 respectively, *P* < 0.0001 by students T test). Normalisation of metrics of heart size to tibia length mitigated all decreases in heart structure, with only a small but significant decrease in heart weight remaining (Fig. 1G–J). This data suggests that although slightly lighter, hearts are otherwise in proportion to body size in *Smn^2B/−^* mice.

**Figure 2:**
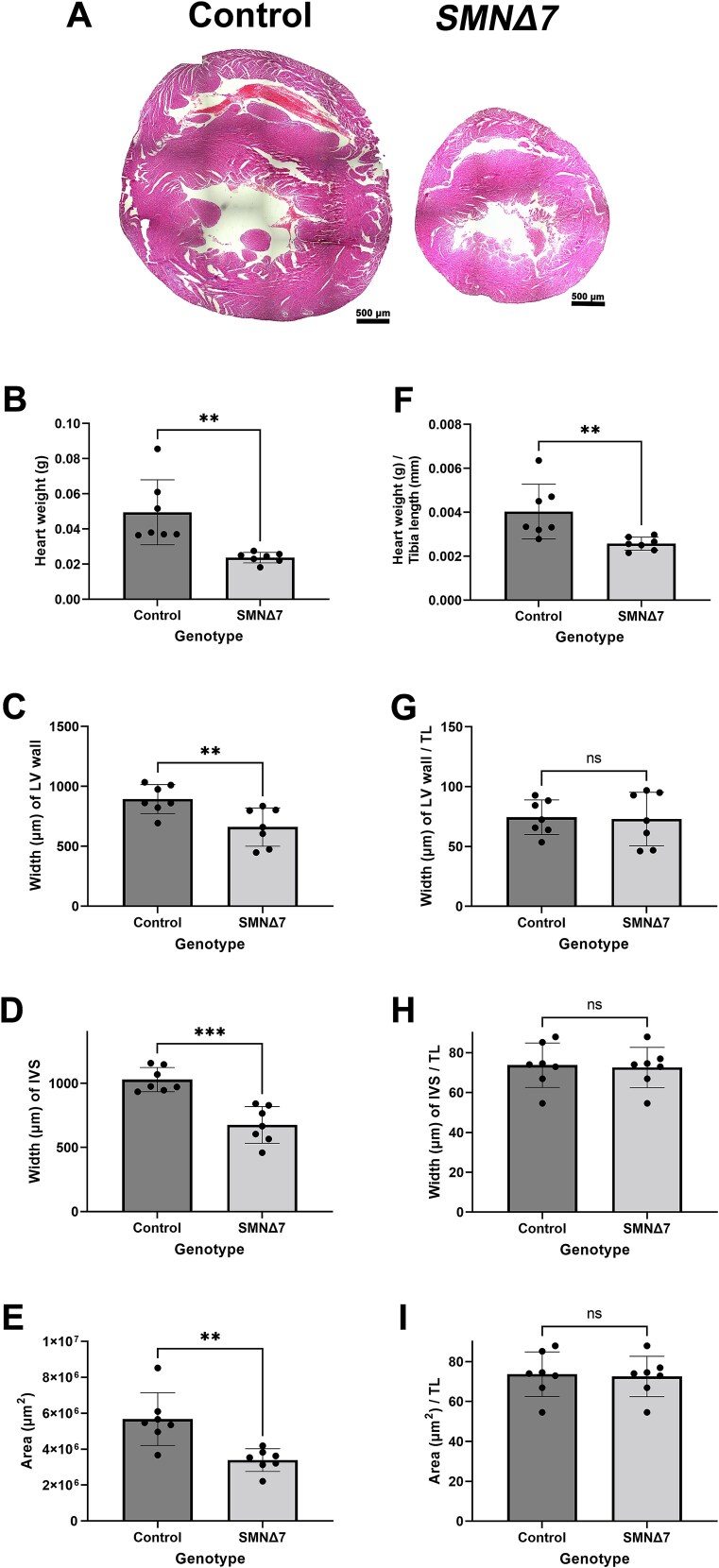
Hearts from endstage *SMNΔ7* mice are lighter but otherwise in proportion to body size. (A) Haematoxylin and eosin stained horizonal sections of hearts at the level of the anterior papillary muscle from endstage *SMNΔ7* mice and healthy littermate controls. Scale bar = 1000 μm. (B–I) Bar charts showing heart weight (B and F), cross sectional area (C and G), left ventricular (LV) wall width (D and H) and width of the interventricular septum (IVS) (E and I) which have been either not normalised (B–E) or normalised to tibia length (TL, F–I). ns, not significant; ^*^^*^P < 0.01; ^*^^*^^*^P < 0.001 by the two tailed unpaired T-test, n = 7 per genotype.

Since some previous studies do not account for body size when measuring dimensions of the heart, to verify the severity of structural changes in the heart in other models, we quantified metrics of heart size in end-stage *SMNΔ7* mice, a severe mouse model of SMA in which heart defects have been consistently reported [12, 17, 19]. Although we observed a similar decrease in all parameters of heart size and wall thickness, once again most defects were mitigated by accounting for body size, other than a small but significant decrease in heart weight (Fig. 2). This work suggests that defects in heart structure can be overstated when body size is not accounted for.

**Figure 3:**
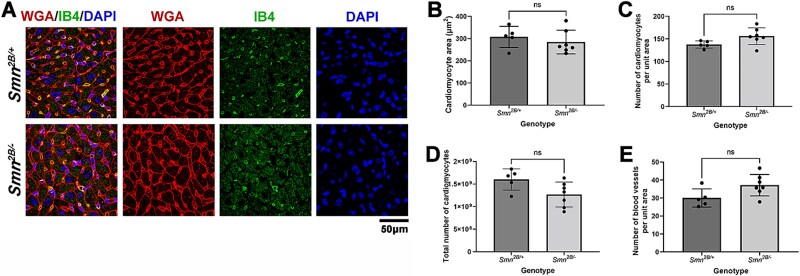
There is no change in cardiomyocyte size or density in the hearts of the *Smn^2B/−^* mouse model at P18. (A) Fluorescent images show heart sections labelled with wheat germ agglutinin (WGA, red), isolectin B4 (IB4; green) and DAPI in P18 *Smn^2B/−^* and *Smn^2B/+^* control mice. Scale bar = 50 μm. (B–E) Bar charts (mean ± SD) showing cardiomyocyte cross sectional area (B), cardiomyocyte density (C), total cardiomyocyte number (D) and blood vessel density (E) in P18 *Smn^2B/−^* and *Smn^2B/+^* control mice. ns, not significant; ^*^^*^P < 0.01; ^*^^*^^*^P < 0.001 by the two tailed unpaired T-test, n = 5 hearts for *Smn^2B/+^* and n = 7 hearts for *Smn^2B/−^*.

To analyse the cellular structure of heart and investigate possible hypertrophy of cardiomyocytes, sections of hearts from *Smn^2B/−^* mice and healthy controls were stained with wheat germ agglutinin to mark cell membranes (Fig. 3A). Quantification of cardiomyocyte cross-sectional area and number of cardiomyocytes (as identified by their distinctive morphology) per unit area revealed no change in *Smn^2B/−^* vs controls (Fig. 3B and C). Since hypertrophy is typically associated with a change in blood vessel density [25], we also quantified blood vessel density using the endothelial cell marker isolectin B4 (IB4) which revealed no difference in blood vessel density (Fig. 3E). Together, these data reveal no overall change in cardiomyocyte size or density. Since altered fibrosis and fat deposition have been previously reported in the hearts of mouse models of SMA, we also evaluated these parameters using picosirius red and Oil-Red-O staining and found no difference in either parameter (Supplementary Fig. 1).

### Echocardiography reveals hyperkinetic changes in the hearts of *Smn^2B/−^* mice at P18.

**Figure 4:**
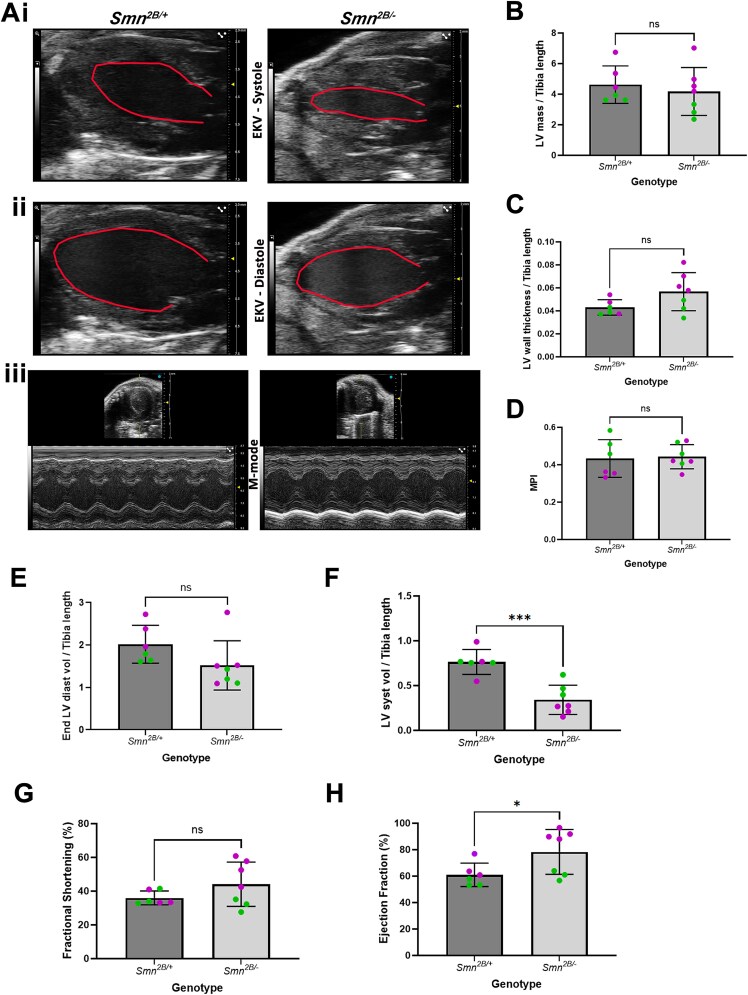
Echocardiography reveals increase systolic function in cohorts of *Smn^2B/−^* mice at P18. (A) EKV (ECG-gated kilohertz visualisation) images of the left ventricle from a parasternal long axis view (i,ii) or short axis view (iii) of the hearts acquired at systole (i) and diastole (ii) or during the cardiac cycle (iii) during echocardiography from the *Smn^2B/+^* (left) and *Smn^2B/−^* (right) genotype. Red line indicates approximate boundary used for measurements of systolic (i) and diastolic (ii) volume (B–H) bar charts showing the normalised left ventricular mass (B), normalised average wall thickness during the cardiac cycle (C), myocardial performance index (D), normalised left ventricular systolic (E) and diastolic (F) volume, fractional shortening (G) and ejection fraction (H) in P18 *Smn^2B/+^* and *Smn^2B/−^* mice. Green data points pertain to litter 1 and magenta data points pertain to litters 2 and 3. ns, not significant; ^*^P < 0.05; ^*^^*^^*^P < 0.001 by the two tailed unpaired T-test, n = 6 hearts for *Smn^2B/+^* and n = 7 hearts for *Smn^2B/−^*.

Since previous work has identified significant perturbations during *in vivo* heart imaging [12, 13, 17], we used high resolution *in vivo* echocardiography to quantify ventricular structure and function in anaesthetised *Smn^2B/−^* mice and *Smn^2B/+^* controls (Fig. 4). To account for the reduced body size in *Smn^2B/−^* mice, all absolute metrics are expressed normalised to tibia length.

For this work, 3 independent litters of mice were evaluated. We observed no significant difference in normalised mass of the left ventricle or average left ventricular wall thickness (Fig. 4A–C), which is consistent with our conclusions from the histological study (c.f. Fig. 1). Myocardial performance index (MPI) was also assessed, which can be used as a marker of global heart failure but no significant difference was observed (Fig. 4D). However, quantification of ventricular volume revealed that while there was no change in diastole, there was a significant decrease in ventricular volume at systole (Fig. 4E and F). As a result of this, there was a trend towards an increase in fractional shortening and significant increase in ejection fraction (Fig. 4G and H).

During the course of this work, it became apparent that there were differences in heart function between litters of *Smn^2B/−^* mice. Whilst function appeared generally preserved in cohort 1 (shown as green data points in fig. 4, EF range 56–64%) there was a dramatic increase in systolic function in cohorts 2 and 3 (shown in magenta in fig. 4, EF range 88%–97%). Indeed, separation of data revealed a significant increase in ejection fraction, fractional shortening, fractional area change and average LV wall thickness in cohorts 2 and 3, but no significant difference in cohort 1 (Supplementary Fig. 2, Supplementary Video 1).

Collectively, the above data suggest that while in subsets of *Smn^2B/−^* mice cardiac function can be preserved, in other cohorts of mice there is a significant increase in systolic function, where a dramatic hyper kinetic phenotype can be observed.

**Figure 5:**
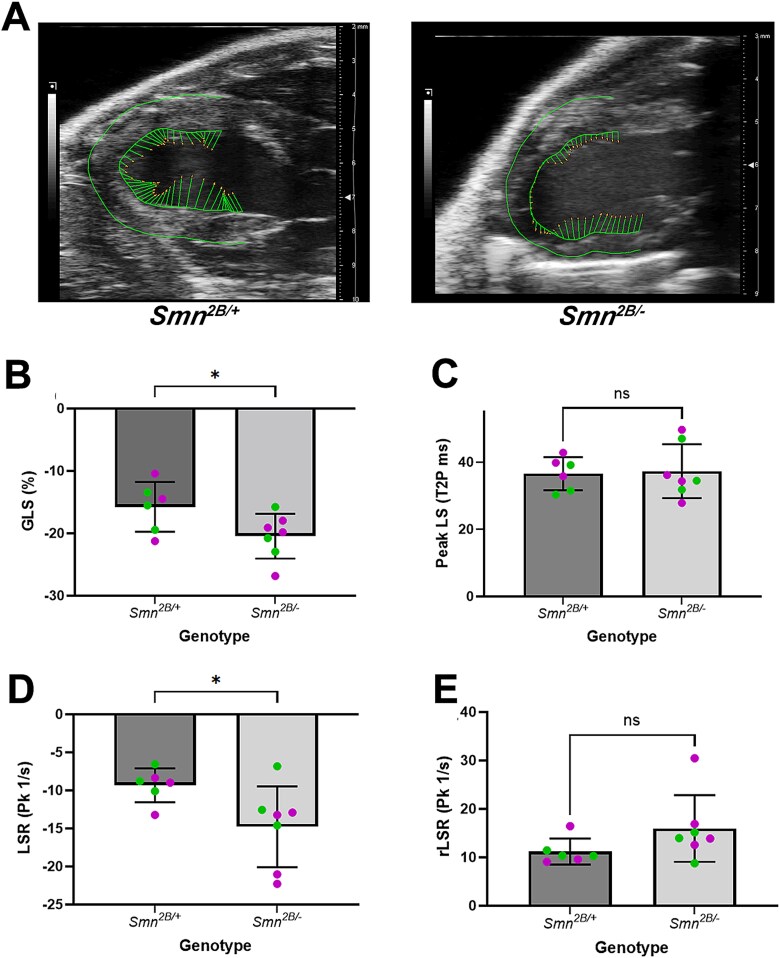
There are abnormalities in global longitudinal strain in the hearts of *Smn^2B/−^* mice at P18. (A) Traced EKV image of the left ventricle from a parasternal long axis view of the hearts acquired during echocardiography with the green lines showing the directional tendencies of every vector in the left ventricular wall trace. (B–E) Bar charts (mean ± SD) showing the global longitudinal strain (GLS; B), peak longitudinal strain (peak LS, C), the longitudinal strain rate (LSR, D) and the reverse longitudinal strain rate (rLSR, E) in *Smn^2B/+^* and *Smn^2B/−^* mice. n = 6 hearts for *Smn^2B/+^* and n = 7 hearts for *Smn^2B/−^*. Green data points pertain to litter 1 and magenta data points pertain to litters 2 and 3. ns: non-significant, ^*^P < 0.05, by the two-tailed unpaired T-test.

Since the above data suggest that subsets of mice show increase systolic function, we undertook 2D speckle tracking strain imaging, which can be more sensitive to changes in ventricular function. Quantification of global longitudinal strain (GLS) revealed a significant increase in *Smn^2B/−^* mice vs controls (*P* = 0.0472) (Fig. 5A and B). There was also an increase in longitudinal strain rate (LSR; i.e. the time taken to reach systolic peak) but no change in peak longitudinal strain (Peak LS) or negative peak (rLSR) (Fig. 5C–E). This data is indicative of subclinical stress within the hearts of *Smn^2B/−^* mice. Such changes can be predictive of an increased risk of heart failure.

### Longitudinal analysis of the proteome reveals a progressive disruption in LXR/RXR signalling

**Figure 6:**
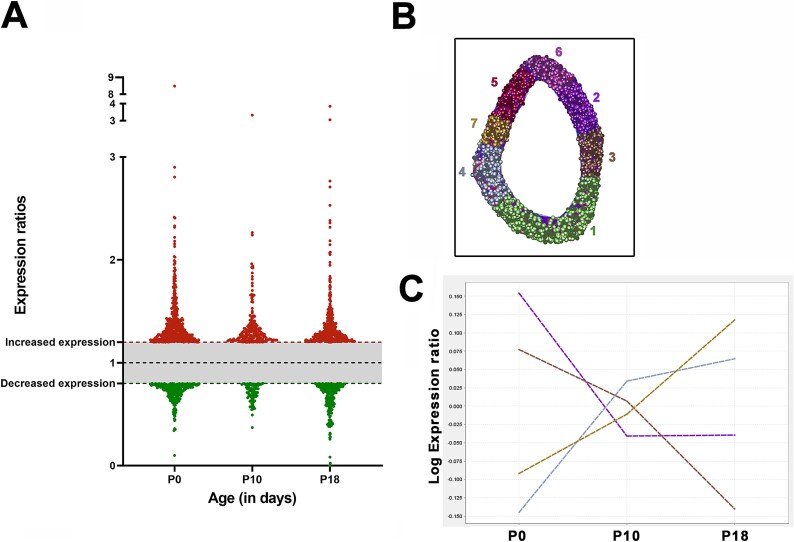
Analysis of the proteome identifies proteins associated with progressive changes over disease time course. (A) Scatterplot representing the proteins which were up (red) or down (green) regulated by 20% or more in *Smn^2B/−^* mice compared to *Smn^2B/+^* mice at P0, P10 and P18. (B) 3D model of protein expression clusters generated using BioLayout 3D identified 7 clusters of expression trends occurring between P0 and P18 in *Smn^2B/−^* mice relative to *Smn^2B/+^* mice. (C) Isolation of the 4 clusters which show changes in relative expression ratio trending upwards or downwards between P0 and P18 which are further analysed below.

The data above indicate that on a gross structural level, hearts dimensions are appropriate to body size in *Smn^2B/−^* mice at disease endstage, but that there are changes in cellular structure and function that results in increased systolic function, accompanied by increase cardiac strain. To gain further insight into the molecular pathways which may underlie these anomalies, we performed proteomic analysis on the hearts of *Smn^2B/−^* mice. In order to identify molecular changes which could contribute to the progression of heart defects, we performed a time course analysis of the proteome of *Smn^2B/−^* and *Smn^2B/+^* at P0, P10 and P18, corresponding with mice being pre-symptomatic, the onset of motor phenotype and endstage, respectively. Tandem mass tagging proteomics identified over 5600 proteins identified by more than 1 unique peptide with between 281 and 716 proteins (at P0 and P10 respectively) showing a change in relative abundance of over 20% in *Smn^2B/−^* mice (Fig. 6A). BioLayoutExpress3D software was used to cluster proteins based upon their relative change in expression compared to that in control hearts over time (Fig. 6B) and proteins which fell into clusters which had a consistent upwards or downward trend between P0 and P18 were isolated (Fig. 6C).

**Figure 7:**
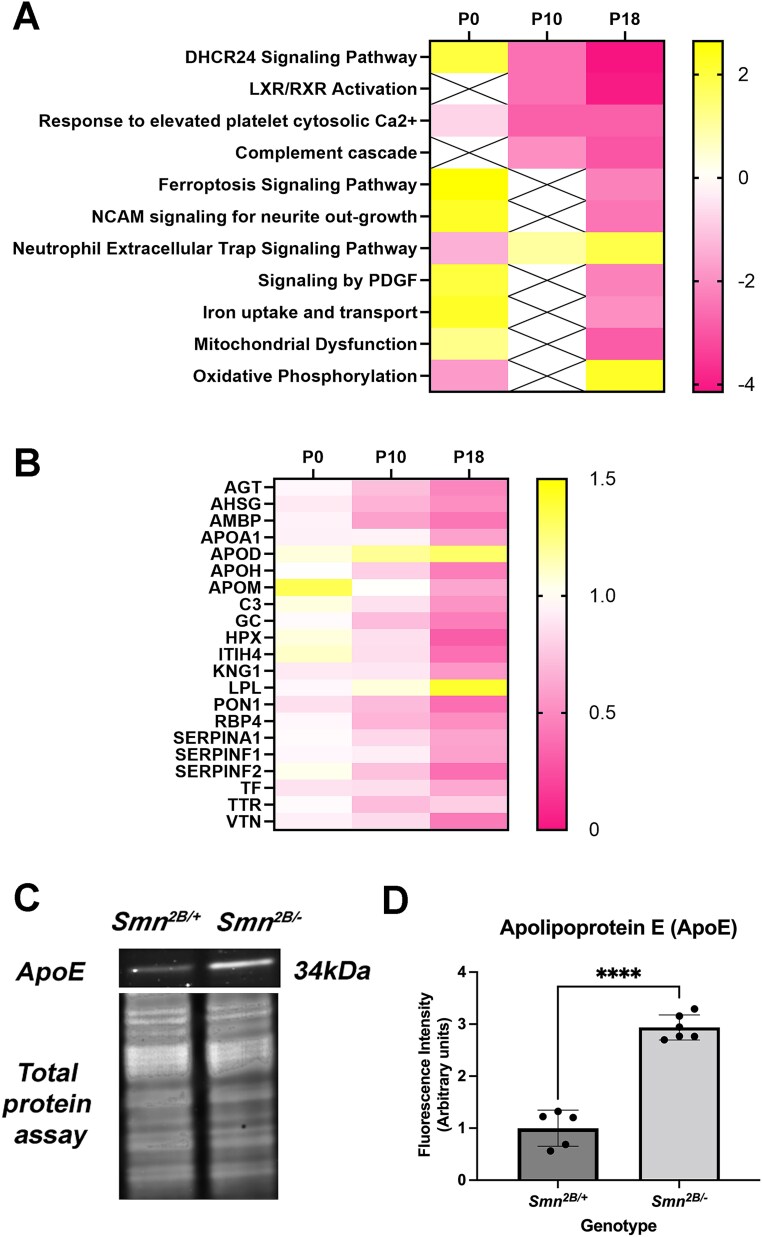
There is a progressive predicted dysregulation of LXR/RXR signalling between P0 and P18. (A) Heat map analysis showing the canonical pathways identified by IPA software ranked by Z score. Heat map denotes Z score at P0, P10 and P18 with a predicted up or down regulation shown in yellow and pink respectively. (B) Heat map denoting relative abundance of proteins which were identified in our data sets pertaining to LXR/RXR signalling at P0, P10 and P18. Relative increase or decrease in relative abundance is shown in yellow and pink respectively. (C and D) Western blot analysis was used to quantify APOE levels in proteins lysate from hearts from P18 *Smn^2B/−^* mice compared to *Smn^2B/+^* controls, expressed normalised to total protein. (mean ± SD; N = 5/6 *Smn^2B/+^/Smn^2B/−^; P* < 0.0001 by Mann Whitney U test.

**Figure 8:**
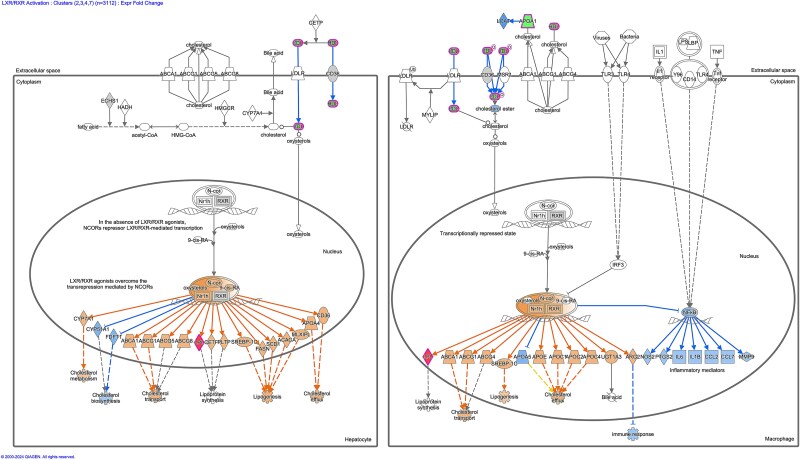
Dysregulation of LXR/RXR signalling pathway at P18 in *Smn^2B/−^* mice. Visual representation of LXR/RXR canonical signalling pathway generated by IPA. Pathway reflects known interactions between proteins as evidenced in hepatocytes and macrophage. This schematic diagram is obtained by conversion of overlaid datasets into fold-change ratios representing expression alteration in the *Smn^2B/−^* mouse model compared to its control using ingenuity pathway analysis. The blue represents predicted downregulation, red represents an upregulation in expression, orange represents a predicted upregulation, grey represents a change less than the 20% threshold, white represents proteins that are involved in the pathway but are not expressed in the dataset.

Ingenuity Pathway Analysis (IPA) software was used to analyse isolated clusters in order to assign functional annotations and identify canonical pathways which are perturbed, with results ranked by z-score (Fig. 7A). This revealed a list of pathways which are progressively dysregulated between P0 and P18 with the highest z scores (and therefore highest predicted activation or inhibition of the pathway) pertaining to DHCR24 signalling and LXR/RXR signalling. Of these, LXR/RXR signalling had the lowest P value and with an activation z score range from 0 at P0, to −2.53 at P10 and to −4.025 at P18. From these results, there was a statistically significant enrichment for LXR-RXR pathway-specific proteins within isolated disease-course-correlative clusters. The directionality of these isolated alterations predicts an inhibition of the pathway activity. We therefore conclude there is a progressive suppression of LXR-RXR signalling between P0 and P18. Heatmap analysis of the proteins which were detected in our screen and are present in LXR-RXR signalling pathways illustrates a progressive alteration in relative expression levels between P0 and P18, with the majority decrease in their relative abundance (Fig. 7B). Liver X receptors (LXR) are nuclear receptors which dimerise with retinoid X receptors (RXR) to regulate expression of their target genes and are known to have a key role in cholesterol and lipoprotein metabolism (Fig. 8). We further verified a significant increase in APO-E levels, a target protein regulated by LXR/RXR signalling, in the heart of P18 *Smn^2B/−^* mice (Fig. 7C and D).

### Multi-model analysis of mouse models of SMA reveals a common disruption in fatty acid metabolism

**Figure 9:**
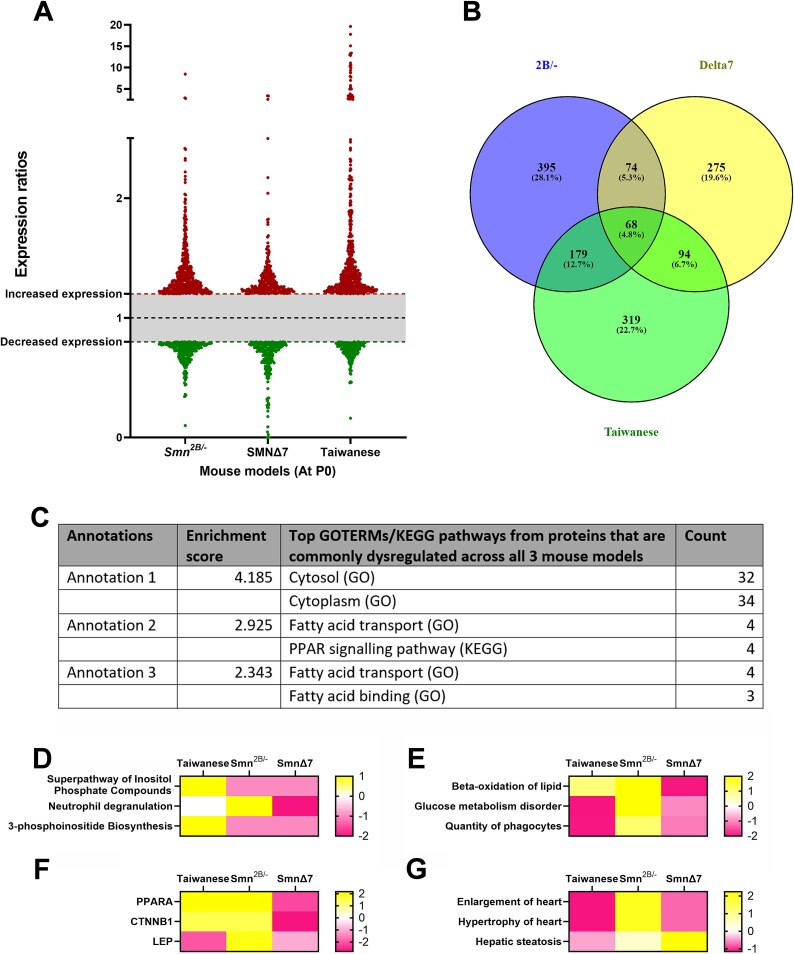
Multi-model analysis reveals common dysregulation of energy metabolism in 3 mouse models of SMA at a pre-symptomatic time point. (A) Scatterplot representing the proteins which were up (red) or down (green) regulated by 20% or more in *Smn^2B/−^*, *SMNΔ7* and Taiwanese mice compared to healthy littermates at P0. (B) Venn diagram showing overlap in dysregulated proteins between three mouse models at P0. (C) Functional clustering of the 68 proteins which were altered in all three mouse models at P0 reveals an enrichment for proteins involved in fatty acid transport and binding. (D–G) Heat maps showing the top 3 canonical pathways (D), molecular toxicity functions (E), up-stream regulators (F) and disease related functions (G) ranked by z-score, as generated by IPA analysis on the 68 proteins which were common across all three mouse models. Note enrichment for pathways, functions and regulators which are involved in fatty acid biosynthesis.

Proteomic data from the *Smn^2B/−^* mouse model is indicative of a perturbation in energy metabolism. In order to determine whether this was a conserved feature across other mouse models of SMA, we performed TMT proteomic profiling in the *Smn^Δ7/Δ7^;SMN2* (aka ‘Taiwanese’) and *Smn^−/−^;Smn^Δ7/Δ7^;SMN2 (aka SMNΔ7*) mouse models of SMA at P0, and compared this proteomic profile to the *Smn^2B/−^* at P0. As before, proteomic profiles were filtered for proteins showing a fold change of greater than 20% compared to their respective healthy control littermate and proteins which were identified by > 1 unique peptide (Fig. 9A). This revealed 511–716 dysregulated proteins, and 68 which were common across all 3 models (Fig. 9B; Supplementary Table 1). Functional clustering analysis using DAVID functional annotation clustering revealed an enrichment for proteins involved in fatty acid transport and binding (Fig. 9C). PPAR signalling was also highlighted, which has important roles in lipid and glucose metabolism. Further analysis of the 68 commonly dysregulated proteins was performed using IPA, and was further indicative of disrupted fatty acid biosynthesis. Specifically, amongst the top three dysregulated canonical pathways, were inositol phosphate biosynthesis (Fig. 9D). Amongst the top three dysregulated disease associated functions were altered beta-oxidation of lipids (Fig. 9E). Analysis of up-stream regulators identified PPARα and Leptin which promote fatty acid catabolism and the regulation of fat storage respectively (Fig. 9F). Analysis of disease associated pathways identified cardiac hypertrophy and hepatic steatosis (Fig. 9G). Collectively, this proteomic analysis points to dysregulated fatty acid biosynthesis in three mouse models of SMA at P0.

## Discussion

Here we show that in the *Smn^2B/−^* mouse model of SMA, heart size is in proportion to body size. We also show that changes in ventricular walls and other aspects of heart structure and function can be overstated when body size is not accounted for. We further show that while cardiac systolic function is increased, but this phenotype is not full penetrant, with subsets of mice presenting with near-normal values and other displaying a dramatically hyperkinetic phenotype. There is an increase in cardiac strain which is indicative of subclinical stress consistent with elevated risk of subsequent heart failure. Proteomic analysis of the heart further reveals a progressive suppression of pathways involved in lipid metabolism. Collectively this work describes the cardiac phenotype in the *Smn^2B/−^* mouse model of SMA, and supports the use of this model to understand the underlying basis of heart pathology in SMA.

### The *Smn^2B/−^* mouse as a model of cardiac pathology in SMA

A first glance the changes in heart size and ventricular wall thickness and changes in cardiac function appeared highly consistent with previously published work that looked at heart pathology in mouse models of SMA [12–14, 17, 19]. However, when body size is accounted for the differences are largely mitigated. We therefore conclude that on a gross structural level, hearts appear proportionate to body size in *Smn^2B/−^* mice. However, more detailed analysis of cardiac function reveals that in a subset of mice, there is an increase in systolic function which suggests hearts in these mice are working harder than is typical. There were also changes in longitudinal strain which are consistent with underlying cardiac stress. The lives of these mice are limited due to their motor phenotype. However, in the event that their life were prolonged, for example by a Smn-upregulating therapy targeted exclusively to the nervous system, it is possible that this underlying stress could develop into cardiac pathology and high risk of heart failure. This phenotype may therefore be very consistent with people with SMA, where there appears to be an increased risk of pathology, but it is not a feature of the disease which is fully penetrant. Indeed, it could be argued that the subclinical cardiac stress observed in the *Smn^2B/−^* mouse model may be more reflective of the human situation, than the more severe and fully penetrant phenotypes observed in more severe mouse models of SMA. Interestingly, changes in global longitudinal strain were recently reported in children with type II and type III SMA [11]. The *Smn^2B/−^* mouse model may therefore be an excellent model to understand the basis for cardiac stress in SMA. However, since the structural and functional defects in the *Smn^2B/−^* mouse model were subtle and not fully penetrant it is equally important to note that for testing strategies to alleviate cardiac pathology in SMA, the more severe models with obvious and easily quantifiable phenotypes may be preferable.

It is notable that the functional perturbations within the model were not fully penetrant, and the most obvious defects were seen in two of the three litters evaluated. All the litters originated from independent breeding pairs and there was no association with litter size. Even in an inbred line such as there, there is the potential for genetic variability and this is perhaps the most likely reason for the non-fully penetrant phenotype. Naturally deciphering the reasons for the variability in phenotype would be extremely valuable, especially due to the parallels in people, where again cardiac pathology is observed only in a subset of people with SMA. Future genetic association studies could be extremely valuable to decipher modifiers of cardiac pathology in SMA and act as important predictors risk of heart defects.

### Importance of body size when considering absolute metrics mouse models

In this study, the interpretation of our data was altered significantly when body size was accounted for. This was true even in the analysis of the *SMNΔ7* mice, where a significant decrease in interventricular septum thickness and left ventricular wall thickness was nullified by normalisation to a surrogate of body size. It is worth noting that the magnitude of change observed in the *SMNΔ7* mice in this study was less than the magnitude of change observed in other studies. This may be explained by inter-colony variations. We therefore acknowledge that important defects in the heart may well exist in other mouse models of SMA, but suggest that these defects can be overstated when the data fails to account for body size.

This consideration is not unique to the heart, and there are numerous instances where absolute measurements are taken but not adjusted for body size, which is frequently decreased in mouse models of neuromuscular diseases. A failure to account for this decrease in size when measuring absolute metrics such as muscle fibre size, motor neuron number or size, muscle cross sectional area, or stride length is evident [26–36]. In our review of the literature, fewer than 10% of studies account for body size when making absolute measurements. This is of critical importance, and there are numerous examples of pre-clinical studies where accounting for body size fundamentally changes the conclusion of the data, frequently turning positive data into negative data.

Whilst accounting for body size may appear intuitive, the reality of accounting for body size rapidly becomes complex. For example, in what way is the mouse smaller: by weight, nose to tail length, or is just part of the body smaller? Does a smaller mouse have fewer cells of the same size, or the same number of smaller cells? The answer is likely to depend on the reason for the smaller body size, and be dependant upon the tissue, cell type, and even the part of the cell which is being considered. Furthermore, the metric that is chosen as a surrogate of body size is highly influential, and selecting a metric which is an accurate reflection of body size and not confounded by other pathological processes is extremely difficult. This work we present here highlights this issue and supports further fundamental research into the relationships between body, tissue and cell size, and how this relates to cell number when body size is decreased in different contexts. This work underlines the importance of considering body size when quantifying any absolute measurement in mouse models and supports the development of methodology to understand how metrics such as muscle fibre size, neuromuscular junction size or motor neuron size are impacted by changes in body size which can occur during postnatal development and/or pathology.

### Disrupted fatty acid metabolism in SMA

Our work supports a progressive disruption of fatty acid metabolism in the hearts of the *Smn^2B/−^* mouse model of SMA, with alterations in the pathways pertaining to fatty acid biosynthesis being evident from the day of birth in 3 mouse models of SMA. We identified a progressive dysregulation of proteins involved in LXR/RXR signalling in the heart of the *Smn^2B/−^* mouse model. Liver X receptors (LXR) are nuclear receptors that, when activated, bind to retinoid X receptors (RXRs) to bind regulatory regions of target genes. In doing so, they influence gene transcription to allow regulation of energy metabolism by influencing cholesterol homeostasis and lipid and glucose metabolism [37]. Our data revealed a progressive activation of LXR/RXR signalling between P0 and P18. Since it is inherently difficult to dissociate changes caused by motor neuron pathology and those which are independent of it, we undertook further analysis of the heart proteome at P0 across three mouse models of SMA. This revealed that on the day of birth, prior to any notable motor neuron loss or motor deficits, there was a consistent dysregulation of proteins involved in fatty acid biosynthesis in the heart. Collectively this data points to a progressive dysregulation in fatty acid metabolism in the heart with defects in biosynthesis being evident prior to any degenerative phenotype, thus supporting the notion that this is a primary defect and not secondary to any motor or cardiac pathology.

Defects in fatty acid metabolism have long been reported in people with SMA and has been reviewed elsewhere [38, 39]. Fatty acids are oxidised to acetyl Co-A, which is then fused to carnitine to form acetyl-carnitine to allow transport into the mitochondria where beta-oxidation of fatty acids can occur [40]. Numerous small studies and case reports on people with SMA have described an increase acetyl-carnitines in urine, a carnitine deficiency in muscle and serum and an increase in the ratio of total to free carnitines in serum [41–43]. A deficiency in acetyl coA dehydrogenase has also been described, pointing to defects in beta-oxidation [41]. People with SMA frequently present with a high fat mass, despite a low caloric intake, pointing to either nutritional problems or defects in the metabolism of fats [39, 44, 45]. Some of the fatty acid defects appear most pronounced in liver, with high proportion of people with SMA displaying a build up of fat in liver cells and fatty vacuoles within the liver [42, 46–49]. This observation has been replicated in mouse models of SMA, wherein the *Smn^2B/−^* mice develop non-alcoholic fatty liver disease, and a change in hepatic tri-glycerides were reported in a range of mouse models of SMA [47]. Interestingly, maintaining *Smn^2B/−^* mice on a low fat diet extended life span [50]. Important, a recent study has reported disrupted lipid profiles in children with type II and III SMA which correlated with changes in global longitudinal strain, indicative of a risk of heart failure [11]. There is therefore extensive evidence that fatty acid metabolism is disrupted both in people with SMA and mouse models of SMA, and clear evidence linking it to the types of phenotype observed in people with SMA.

The observed change in fatty acid metabolism can indeed be indicative of a primary fatty acid oxidation disorder, but is also often commonly seen as a secondary defect in neuromuscular disorders. Indeed historically it has been assumed that the carnitine linked anomalies in people were secondary to the loss in muscle mass occurring in SMA. However, more recent evidence is indicative of a primary lipid metabolism defect. Indeed, hepatocytes derived from patient iPSCs display increase lipid accumulation, increase ATP and succinate dehydrogenase activity [49]. Proteomic analysis of these cells revealed a decrease in oxidative phosphorylation and lipid metabolism [49]. This is supportive of our own data, we observe disruptions in fatty acid biosynthesis across 3 mouse models on the day of birth, which could therefore not be secondary to any motor neuron loss or muscle atrophy. Data from our lab and others is therefore supportive of a primary defect in the metabolism of fatty acids in SMA.

Many of the changes which we report within this study bear striking similarities to the molecular and functional changes reports in diabetic cardiomyopathy. The pathophysiology of diabetic cardiomyopathy is complex, and often confounded by other comorbidities, but it is recognised that there is a change in energy substrate usage within the heart, with an increased reliance on fatty acids as an energy source over glucose [51]. As fatty acid oxidation rates increase, there is an increased in oxygen consumption and increase in PPARα signalling which is linked to the downstream cardiac dysfunction [51, 52]. Similar change in entergy substrate usage and PPARα signalling were noted in this study in the SMA mouse model hearts. Interestingly, in a large retrospective study of people with diabetic cardiomyopathy, changes in global longitudinal strain appear to precede other changes in systolic and diastolic function [53], which is again a functional parameter which was altered in our study. These results therefore bear striking correlation to our own, wherein changes in energy metabolism and increase in PPARα signalling was also associated with an increase in global longitudinal strain. Indeed, LXR pathway agonists have shown the ability to reduce oxidative stress and attenuate myocardial dysfunction in a diabetic rat model [54]. This raises the intriguing possibility that some of the molecular changes that we are observing in the heart are actually compensatory and protective in the hearts of the mouse models.

Based upon the correlation with the aetiology and presentation in diabetic cardiomyopathy, we suggest that the changes in fatty acid metabolism and global longitudinal strain are predicative of a subsequent high risk of cardiac failure and provide an explanation for the high incidence of cardiac defects which are routinely observed in people with SMA. In conclusion, this work highlights the need for further research into the defects in energy metabolism in SMA, how they link to the high risk of cardiac problems in SMA and importantly, how this risk can be mitigated. We suggest that targeting metabolic dysfunction in SMA could have wide ranging benefits, including lowering the risk of cardiovascular complications in SMA.

## Supplementary Material

Supplementary_Figures_ddaf006

supplementary_table_1_ddaf006

Supplementary_video_1_ddaf006

Supplementary_figure_legends_ddaf006
