# Supplementary material for: Alterations in cardiac function correlate with a disruption in fatty acid metabolism in a mouse model of SMA
Source: Hum Mol Genet. 2025 Jan 15;34(6):547–62. doi: 10.1093/hmg/ddaf006 (PMC11891873; doi:10.1093/hmg/ddaf006)
Supplement: Supplementary_Figures_ddaf006 [file supplementary_figures_ddaf006.pdf]

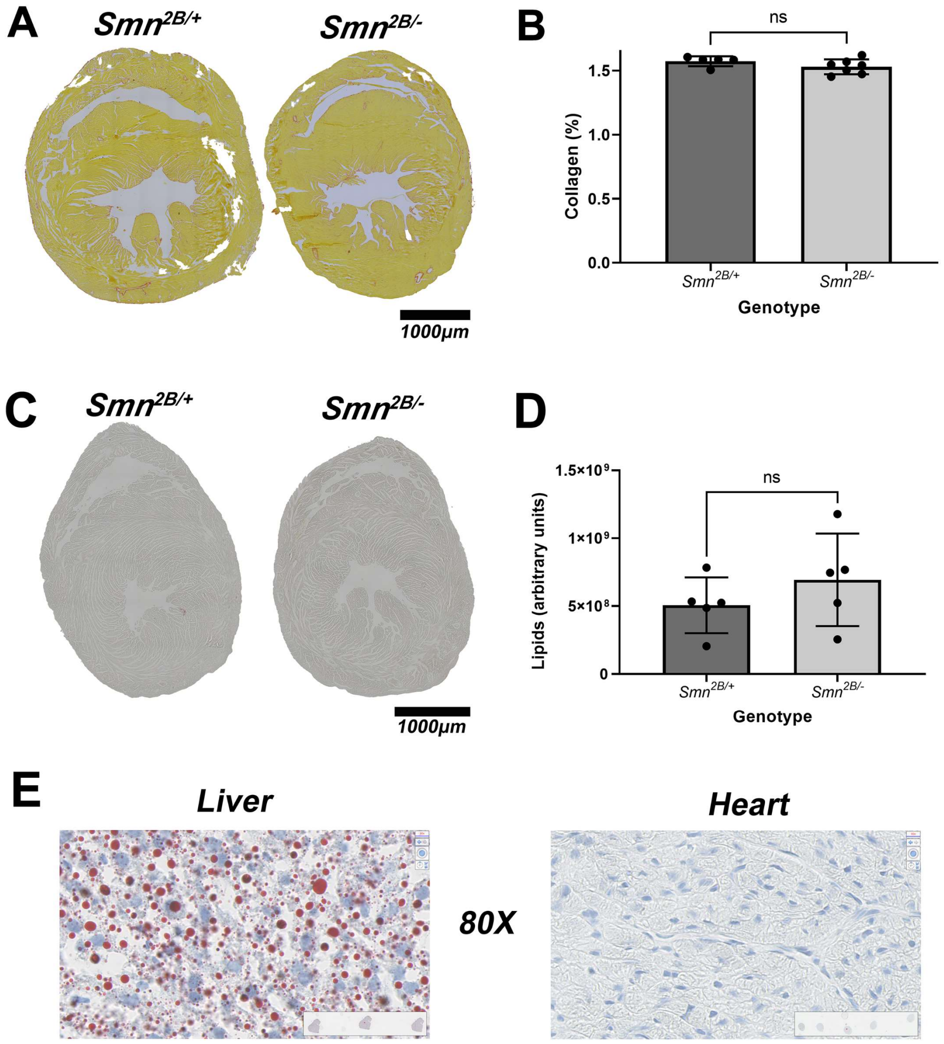

Supplementary Figure 1: No evidence of fibrosis or fat accumulation in the hearts of Smn2B/- mice. A,C) Images showing sections of heart from P18 Smn2B/+ or Smn2B/- mice stained with Picosirius red (total fibrillar collagen, A) or Oil Red-O (lipid, C). B,D) Bar charts (Systolic and diastolic function differ between cohorts in P18 *Smn$^{2B/-}$* mice. (A) EK Figure 3: There is no change in cardiomyocyte size or density in the hearts of the *Smn$^{2B/-}$* mouse model at P18. B, D) Bar charts (Mean ± SD) showing no significant difference in the percentage of the section which is positive for picosirius red (B) or the raw integrated density of Oil-Red-O staining (D) between Smn2B/+ and Smn2B/- hearts. E) Since very little positive staining was seen for Oil-Red-O in cardiac tissue, we verified protocols using liver tissue and show clear staining of lipid droplets within the liver but an absence of staining in the P18 hearts. ns, not significant; the two tailed unpaired T-test. For picosirius red n=5/7 hearts for *Smn$^{2B/+}$* /*Smn$^{2B/-}$* . For Oil-Red-O n=5/5 hearts for *Smn$^{2B/+}$* /*Smn$^{2B/-}$*.

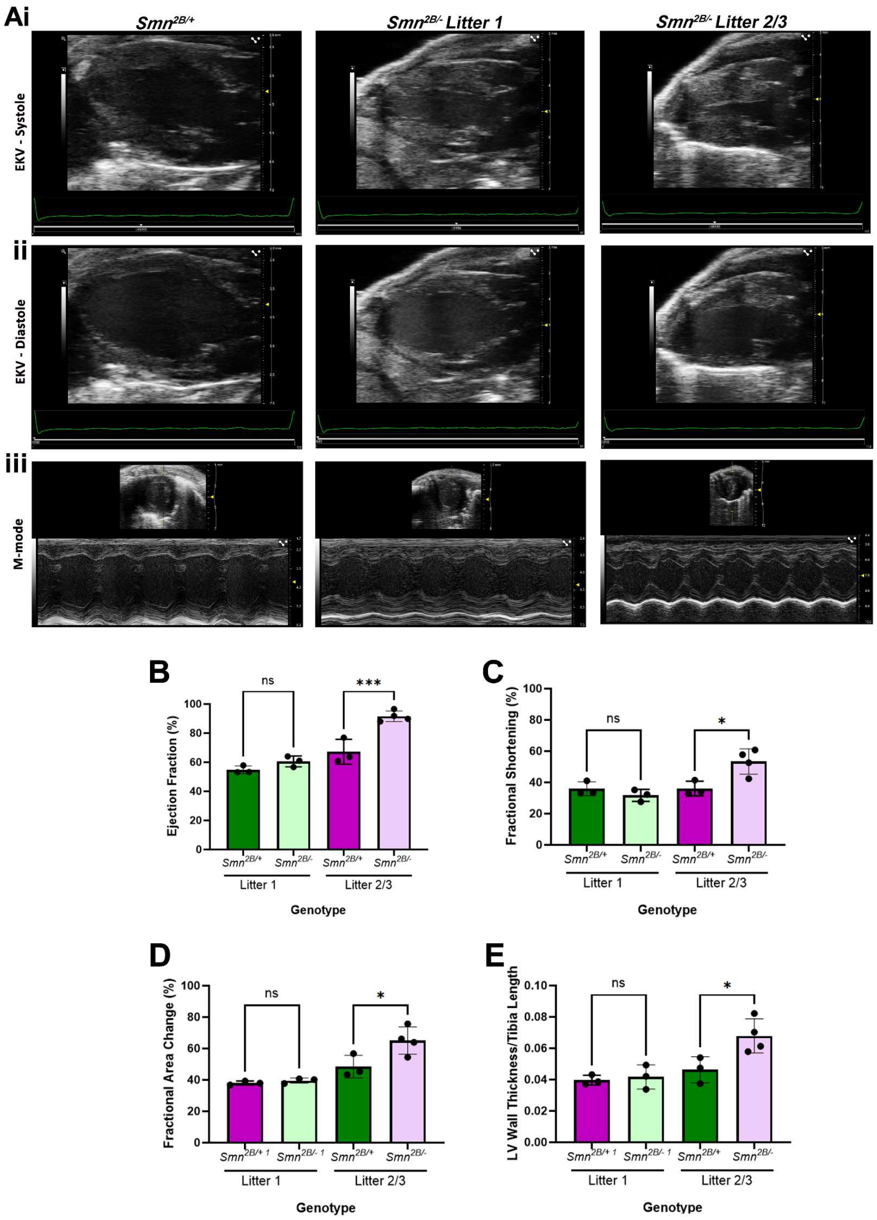

Supplementary Figure 2: Perturbations in systolic function in subsets of *Smn*$^{2B/-}$ mice. (A) EKV (ECG-gated Kilohertz Visualisation) images of the left ventricle from a parasternal long axis view (i,ii) or short axis view (iii) of the hearts acquired at systole (i) and diastole (ii) or during the cardiac cycle (iii) during echocardiography from the *Smn*$^{2B/+}$ (left), *Smn*$^{2B/-}$ litter 1 (middle) and *Smn*$^{2B/-}$ litter 2/3 (right) genotype, (B-E) Bar charts showing the ejection fraction (B), fractional shortening (C), fractional area change (D), and normalised average wall thickness during the cardiac cycle (E) in P18 *Smn*$^{2B/+}$ and *Smn*$^{2B/-}$ mice which are from litter 1 or litters 2/3. ns, not significant; ***P<0.001; * P<0.05; ns non significant by Anova with Tukey multiple comparison test. n=3/3 hearts for *Smn*$^{2B/+}$ for litters 1 or 2/3 respectively, n=3/4 hearts for *Smn*$^{2B/-}$ for litters 1 or 2/3 respectively.
